# Melatonin–iron oxide nanoparticles synergy enhances salt tolerance in *Mentha × piperita* L.

**DOI:** 10.1080/15592324.2026.2657091

**Published:** 2026-04-16

**Authors:** Tauseef Anwar, Huma Qureshi, Hira Ayub, Hossam S. El-Beltagi, Salohiddinjon Yunusov, Shuqurillo Ziyadov, Muydinjon M. Muminov, Gamal Awad El-Shaboury, Dilbar Bazarbayeva, Ibtisam M. Alsudays, Khalid H. Alamer, Munisa Bekmukhamedova, Bakhrom Jobborov, Mohd Asif Shah

**Affiliations:** a Department of Botany, The Islamia University of Bahawalpur, Bahawalpur, Pakistan; b Department of Botany, University of Chakwal, Chakwal, Pakistan; c Agricultural Biotechnology Department, College of Agriculture and Food Sciences, King Faisal University, Al-Ahsa, Saudi Arabia; d Department of Horticulture and Viticulture, Tashkent State Agrarian University, Tashkent region, Uzbekistan; e Department of Ecology, National University of Uzbekistan named after Mirzo Ulugbek, Tashkent, Uzbekistan; f Department of Chemistry, Andijan State University, Andijan, Uzbekistan; g Department of Startup Projects, Kokond University Andijan Branch, Andijan, Uzbekistan; h Biology Department, College of Science, King Khalid University, Abha, Saudi Arabia; i Department of Engineering Technology and Environmental Protection, Nukus State Technical University, Nukus, Uzbekistan; j Department of Biology, College of Science, Qassim University, Qassim, Saudi Arabia; k Biological Sciences Department, Faculty of Science and Arts, King Abdulaziz University, Rabigh, Saudi Arabia; l Department of Ecology and Hydrogeology, University of Geological Sciences, Tashkent, Uzbekistan; m Kardan University, Kabul, Afghanistan; n Division of Research and Development, Lovely Professional University, Phagwara, India; o University Center for Research & Development, Chandigarh University, Mohali, India

**Keywords:** Photosynthetic pigments, lipid peroxidation, electrolyte leakage, biogenic nanomaterials, reactive oxygen species, nano-biostimulants

## Abstract

Iron is an essential micronutrient involved in chlorophyll biosynthesis, redox metabolism, and antioxidant enzyme function, and its availability becomes particularly important under salinity stress. This study evaluated the individual and combined effects of green-synthesized iron oxide nanoparticles (IONPs) and melatonin on the salt stress response of *Mentha × piperita* L. A controlled greenhouse experiment was conducted using a three-factor factorial design with melatonin (0 and 0.1 g L^−1^), IONPs (0 and 0.5 g L^−1^), and NaCl (0 and 100  mM). Treatments were applied as foliar sprays for four weeks, and plant growth traits, photosynthetic pigments, oxidative damage markers, membrane stability, and antioxidant enzyme activities were assessed. Salinity markedly reduced leaf, shoot, and root growth, decreased the chlorophyll content, and increased hydrogen peroxide, malondialdehyde, and electrolyte leakage. In contrast, melatonin and IONPs alleviated these adverse effects, with the combined treatment producing the strongest response. Under saline conditions, the combined application of IONPs and melatonin increased shoot and root growth, improved chlorophyll retention, reduced oxidative damage, and enhanced the activities of superoxide dismutase, peroxidase, and catalase relative to NaCl-treated plants. These results indicate that the combined treatment improved salt tolerance by strengthening antioxidant defense, preserving membrane integrity, and sustaining photosynthetic performance. These findings suggest that biogenic iron oxide nanoparticles, particularly when integrated with melatonin, may serve as effective nano-biostimulant tools for improving peppermint resilience under salinity stress. This study provides a physiological and biochemical basis for the use of iron-based nanomaterials and plant bioregulators in the management of salt-affected cultivation systems.

## Introduction

Abiotic stresses, particularly salinity, are among the most serious constraints on agricultural productivity worldwide because they inhibit plant growth, reduce yield, and disrupt physiological and metabolic processes.^1^ Salinity stress exerts its harmful effects through osmotic imbalance, ion toxicity, nutrient disequilibrium, and oxidative damage, ultimately impairing plant development and crop quality.^2^^,^^3^ In many plant species, excessive Na⁺ accumulation occurs initially in the roots and it is subsequently transported to stems and leaves, where it disturbs cellular homeostasis, damages membranes, interferes with K⁺ uptake, and impairs enzyme functioning.^4^ In crops such as rice, wheat, and tomato, Na⁺ accumulation in root and leaf tissues is closely associated with reduced chlorophyll content, impaired stomatal regulation, and decreased biomass production.^5^^,^^6^ Because of the increasing extent of salt-affected soils, there is a pressing need for innovative and sustainable approaches that improve plant tolerance to salinity without compromising productivity. In this context, nanomaterials and plant growth regulators have emerged as promising tools for enhancing stress resilience.^7^^,^^8^ Among them, iron-based nanoparticles and melatonin have received considerable attention because of their ability to improve nutrient acquisition, regulate stress-responsive pathways, and strengthen antioxidant defense systems. Although both agents have shown beneficial effects individually under saline conditions, their combined action remains poorly understood, especially in medicinal and aromatic plants such as *Mentha × piperita* L. (peppermint). Given the economic importance of peppermint as an essential-oil-producing crop,^9^ clarifying the physiological basis of its response to these treatments under salinity is essential for the development of sustainable cultivation strategies in salt-affected regions.

Peppermint, a hybrid of *Mentha aquatica* L. and *Mentha spicata* L., is widely cultivated for its essential oil, which is rich in biologically active constituents such as menthol, menthone, and menthyl acetate.^9^^,^^10^ These compounds are highly valued in the pharmaceutical, food, cosmetic, and flavor industries because of their antimicrobial, antioxidant, and therapeutic properties.^11^^,^^12^ However, peppermint is considered sensitive to salinity, and salt stress markedly reduces its biomass, essential oil yield, and overall physiological performance.^13^ Salinity can alter photosynthesis, nutrient uptake, stomatal behavior, and enzymatic metabolism in peppermint, while also affecting the composition of secondary metabolites that determine its commercial quality.^14^ At the tissue level, salinity-induced accumulation of toxic ions, especially Na⁺, disrupts leaf metabolism, reduces chloroplast efficiency, and limits carbon assimilation and growth.^15^ Askary et al.^16^ reported that iron nanoparticles influenced the response of *M. piperita* under salinity stress, highlighting the importance of iron-based nanomaterials for improving peppermint performance under adverse environmental conditions. Therefore, improving salt tolerance in peppermint is important not only for maintaining biomass production but also for preserving oil quality and economic value.

Melatonin, a naturally occurring indoleamine, has emerged as a central regulator of plant adaptation to environmental stresses, including salinity. Although first identified in animals, melatonin is now recognized as a multifunctional molecule in plants, where it participates in growth regulation, redox homeostasis, and stress signaling.^17^ Under salt stress, melatonin acts as a powerful antioxidant by directly scavenging reactive oxygen species (ROS) and by stimulating enzymatic antioxidant systems, including superoxide dismutase, peroxidase, and catalase.^18^ In several plant species, melatonin treatment has been associated with enhanced activities of these antioxidant enzymes in specific tissues, reduced hydrogen peroxide and malondialdehyde accumulation, and improved membrane stability.^19^^,^^20^ For example, in rice leaves, melatonin has been shown to enhance SOD and CAT activity under salinity, thereby protecting the photosynthetic apparatus.^21^ In tomato seedlings, melatonin improved POD and CAT activities while reducing lipid peroxidation in both root and leaf tissues.^22^ Similarly, in wheat, melatonin-mediated activation of antioxidant metabolism has been linked with improved osmotic balance and reduced oxidative damage under salt stress.^23^ Moreover, melatonin contributes to ion homeostasis by regulating Na⁺ and K⁺ transport, thereby limiting toxic Na⁺ accumulation in roots and leaves and improving selective ion uptake under saline conditions.^24^ Melatonin also interacts with phytohormonal and signaling networks to promote osmotic adjustment, maintain chlorophyll content, and sustain photosynthetic activity.^25^ Giglou et al. demonstrated in peppermint that exogenous melatonin, under simultaneous exposure to chitosan-coated Fe_3_O_4_ nanoparticles, differentially affected biomass, total carbohydrates, and essential oil production, further emphasizing the relevance of melatonin-based interventions in this medicinal plant.^26^ Despite these advances, the physiological and biochemical roles of melatonin in peppermint under saline conditions remain insufficiently characterized.

Nanotechnology has likewise emerged as an effective strategy to improve plant nutrition and tolerance to abiotic stress. Among different nanomaterials, iron oxide nanoparticles (IONPs) are particularly promising because iron is an essential micronutrient involved in chlorophyll biosynthesis, electron transport, respiration, and the activity of many enzymes.^27^ Under saline conditions, iron availability and uptake are often reduced, which can intensify chlorosis, oxidative stress, and metabolic dysfunction. Owing to their high surface area, reactivity, and bioavailability, IONPs may improve Fe acquisition more efficiently than conventional sources and thereby support metabolic functions under stress. In addition, IONPs have been reported to stimulate antioxidant defense, improve photosynthetic performance, and enhance water relations in plants exposed to salinity.^28^ Studies in crops such as maize, wheat, and soybean have shown that iron nanoparticles can reduce ROS accumulation, improve chlorophyll formation, and enhance stress tolerance under saline environments.^29^ Iron nanoparticles can modulate plant performance under salinity stress.^16^ However, despite these promising findings, the use of IONPs in medicinal and aromatic plants remains comparatively underexplored, and their physiological role under salt stress requires further clarification.

Recent evidence suggests that the interaction between melatonin and nanoparticles may provide a complementary or even synergistic strategy for stress mitigation.^30^ Therefore, the present study was designed to evaluate the individual and combined effects of IONPs and melatonin on the growth, physiological traits, and antioxidant enzyme activities of peppermint under both normal and saline conditions. It was hypothesized that the combined treatment would provide greater protection than either factor alone by enhancing antioxidant defense, maintaining ion homeostasis, and preserving photosynthetic efficiency. Elucidating these responses will improve our understanding of how nanomaterials and plant bioregulators can be integrated to support sustainable peppermint cultivation in saline environments.

## Materials and methods

### Experimental location, duration, and greenhouse conditions

This study was conducted during 2023 as a single-season controlled greenhouse experiment to evaluate the individual and combined effects of melatonin and plant-mediated iron oxide nanoparticles (IONPs) on the salt stress response of peppermint. The experiment was carried out at the Department of Botany, The Islamia University of Bahawalpur, Pakistan (29°24′0″ N, 71°41′0″ E). The region is characterized by an arid to semi-arid climate, which is suitable for salinity-related plant studies. The experiment was performed only in 2023 and was not repeated in subsequent years. However, because it was conducted under greenhouse conditions, the main environmental variables, including temperature, humidity, irrigation, and photoperiod, were regulated throughout the study, thereby minimizing seasonal and geographical variation. The greenhouse day/night temperatures were maintained at 25–30 °C and 18–22 °C, respectively, and the relative humidity ranged from 55% to 65%. The plants were grown under a 16 h/8 h light/dark photoperiod. The natural light was supplemented with artificial lighting to maintain a uniform photoperiod and to minimize variation in plant exposure across treatments and replicates. The total light intensity at the canopy level was 450 µmol m^–2^ s^–1^, of which natural light contributed approximately 300 µmol m^–2^ s^–1^, and supplemental artificial light contributed approximately 150 µmol m^–2^ s^–1^.

### Plant material and establishment

Healthy peppermint plants were obtained from the Botanical Garden, Department of Botany, The Islamia University of Bahawalpur, Pakistan. The cultivar/variety used in the present study was local peppermint stock maintained in the Botanical Garden. At the time of transplantation, plants were approximately 30 d old and had a mean height of 10.5  cm. Uniform and healthy planting material was selected for transplantation to minimize variation among experimental units. Before transplanting, roots were washed thoroughly with tap water followed by distilled water to remove adhering soil particles.

Each plant was grown in a 5 kg plastic pot provided with a drainage hole to prevent waterlogging. The growth medium consisted of sandy loam soil. Before planting, the soil was air-dried, passed through a 2 mm sieve, and sterilized to reduce possible contamination. Initially, three plants were established in each pot, and they were later thinned to one plant per pot to ensure uniform growth and to avoid competition among plants. Irrigation was carried out regularly using distilled water, except during the salinity treatment period, and weeds were removed manually when necessary.

### Soil characteristics and salinity background

Before treatment application, the soil had a pH of 7.2, an electrical conductivity (EC) of 1.3 dS m^–1^, and an organic matter content of 1.2%. Salinity stress was imposed using sodium chloride (NaCl) as the sole salt source. The initial total dissolved solids (TDS) of the soil were 832 mg L^–1^. Soil physicochemical properties were assessed before and after treatment application to confirm the salinity status of the substrate and document changes associated with NaCl exposure.

After treatment application, the final soil pH, EC, and TDS were recorded as 7.6, 5.8 dS m^–1^, and 3712  mg L^−1^, respectively. These measurements were used to verify that the imposed treatments altered the soil environment in the intended manner.

### Synthesis and characterization of iron oxide nanoparticles

Fresh leaves of *Moringa oleifera* were collected from the Botanical Garden of the Department of Botany, The Islamia University of Bahawalpur, Pakistan (29°24′0″ N, 71°41′0″ E). The collection was carried out with departmental permission, and a voucher specimen (IUB/BOT/2598635748) was deposited in the departmental herbarium. The leaves were washed thoroughly with distilled water, chopped into small pieces, and used to prepare the plant extract. *M. oleifera* was selected for nanoparticle synthesis because its leaves are rich in polyphenols, flavonoids, and related phytochemicals that can function as natural reducing and capping agents.^31^

For extract preparation, 30 g of fresh leaves was mixed with 100 mL of distilled water and heated at 60 °C for 30 min. The mixture was then filtered through Whatman No. 1 filter paper, and the filtrate was stored at 4 °C for further use. The leaf extract was used as a natural reducing and stabilizing agent for the green synthesis of IONPs. A 0.1 M FeCl₃ solution was prepared, and the extract was added dropwise under constant stirring until visible precipitation indicated nanoparticle formation.

The synthesized IONPs were characterized to determine their structural, chemical, and physicochemical properties. The crystalline structure was analyzed using a GBC EMMA X-ray diffractometer, where GBC EMMA refers to the instrument model used for X-ray diffraction-based phase identification and crystal structure analysis. The surface morphology was examined by scanning electron microscopy (SEM), elemental composition was verified by energy-dispersive X-ray spectroscopy (EDX), and Fourier-transform infrared (FTIR) spectroscopy was used to identify surface functional groups associated with nanoparticle stabilization. Optical properties were assessed by UV-Vis spectroscopy. In addition, the zeta potential, hydrodynamic diameter, and suspension stability of the synthesized IONPs were determined. The zeta potential was -24.6 mV, the hydrodynamic diameter was 118.4 nm, and the nanoparticle suspension remained moderately stable for 72 h.

### Experimental design and treatment application

The experiment followed a completely randomized design with a three-factor factorial arrangement involving melatonin, iron oxide nanoparticles, and salinity stress. The three independent factors were melatonin at 0 and 0.1 g L^–1^, IONPs at 0 and 0.5 g L^–1^, and salt stress at 0 and 100  mM NaCl. Each treatment combination was replicated three times, with one plant maintained per pot after thinning, resulting in a total of 24 experimental units.

The study was specifically designed to assess the role of IONPs and melatonin, applied individually and in combination, in modulating the salt-mediated stress response of peppermint. The evaluated parameters included growth traits, photosynthetic pigment content, oxidative damage markers, and the activities of the major antioxidant enzymes superoxide dismutase (SOD), peroxidase (POD), and catalase (CAT). Thus, the primary aim was to determine whether these iron-based nano-biostimulant treatments could improve peppermint performance under salt-affected conditions.

The concentrations of melatonin (0.1 g L^–1^) and IONPs (0.5 g L^–1^) were selected based on previous reports demonstrating the efficacy of exogenous melatonin and iron oxide nanoparticles in improving plant performance under salinity stress, as well as on their suitability as biologically active doses for an initial factorial evaluation.^32^ Although only one concentration of melatonin and IONPs was used in the present study, these levels were selected as effective working doses for evaluating treatment performance under saline conditions. It is acknowledged, however, that the use of a single dose does not allow full assessment of optimal treatment levels, dose-response relationships, or potential nanoparticle toxicity. These aspects should be addressed in future studies using multiple treatment concentrations.

Treatments were applied after plants had completed 30 d of growth. Melatonin and IONPs were administered as foliar sprays at 7-day intervals for four weeks. In the combined treatment, melatonin and IONPs were applied simultaneously as foliar sprays. Salt stress was imposed gradually by applying NaCl solution in increments of 25  mM every 2 d until the final concentration of 100  mM was reached to avoid osmotic shock.

### Morphological and physiological measurements

Morphological and physiological parameters were recorded after 4 weeks of treatment application.

### Morphological and growth parameters

Leaf area was measured using a leaf area meter (LI-3100C, USA) from fully expanded leaves collected from the second node from the apex. Shoot length and root length were measured using a measuring scale. Fresh biomass and dry biomass were recorded using an electric balance. The dry weight was determined by oven-drying the samples at 70 °C until constant weight was achieved.

### Electrolyte leakage, oxidative stress markers, and chlorophyll contents

Electrolyte leakage was determined by placing 1 g of fresh leaf tissue in 10 mL of distilled water and incubating the samples at room temperature for 24 h. The initial electrical conductivity (EC₁) was measured using a conductivity meter. The same samples were then boiled for 30 min, cooled to room temperature, and the final conductivity (EC₂) was recorded. Electrolyte leakage was calculated as follows^33^:

EL(%)=(EC₁/EC₂)×100

Oxidative stress markers, including malondialdehyde (MDA) and hydrogen peroxide (H₂O₂), were quantified spectrophotometrically.^34^ The MDA content was calculated using the following equation:

MDA(µmol g⁻¹ FW)=6.45(A₅₃₂−A₆₀₀)−0.56A₄₅₀

H₂O₂ content was estimated from absorbance at 390 nm using a standard calibration curve:

H₂O₂(mmol g⁻¹ FW)=A₃₉₀/standard curve slope

Chlorophyll pigments were extracted using 80% acetone and measured with a UV-Vis spectrophotometer at 663 nm for chlorophyll *a* and 645 nm for chlorophyll *b.*^35^ Chlorophyll concentrations were calculated as follows:

$$\mathrm{Chlorophyll a}(\mathrm{mg g}⁻¹)=((12.7\times A_{663})-(2.69\times A_{645}))\times V/(1000\times W)$$

$$\mathrm{Chlorophyll b}(\mathrm{mg g}⁻¹)=((22.9\times A_{645})-(4.68\times A_{663}))\times V/(1000\times W)$$

Total chlorophyll(mg g⁻¹)=Chlorophyll a+Chlorophyll b

where A₆₆₃ and A₆₄₅ are the absorbance values at 663 and 645 nm, respectively, V is the extract volume (mL), and W is the fresh weight of the sample (g).

#### Antioxidant enzyme assays

Antioxidant enzyme activities were measured to evaluate the biochemical response of peppermint plants to salinity and exogenous treatments. For enzyme extraction, 0.5 g fresh leaf tissue was homogenized in 5 mL ice-cold 50 mM phosphate buffer (pH 7.0) containing 1 mM EDTA and 1% polyvinylpyrrolidone (PVP). The homogenate was centrifuged at 12,000 × g for 20 min at 4 °C, and the resulting supernatant was used immediately for enzyme assays.

Superoxide dismutase (SOD; EC 1.15.1.1) activity was determined by measuring the inhibition of nitroblue tetrazolium (NBT) photoreduction at 560 nm.^36^ The reaction mixture contained 50  mM phosphate buffer (pH 7.8), 13 mM methionine, 75 µM NBT, 2 µM riboflavin, 0.1 mM EDTA, and an enzyme extract. The reaction tubes were illuminated to initiate photoreduction, and the absorbance was recorded at 560 nm. One unit of SOD activity was defined as the amount of enzyme required to produce 50% inhibition of NBT photoreduction under the assay conditions.

Peroxidase (POD; EC 1.11.1.7) activity was assayed using 4-methyl catechol as the substrate by monitoring the increase in absorbance at 420 nm.^37^ The reaction mixture consisted of 50  mM phosphate buffer (pH 6.0), 4-methyl catechol, H₂O₂, and the enzyme extract. After the addition of the enzyme extract, the change in absorbance was recorded for 1  min, and activity was calculated from the rate of substrate oxidation.

Catalase (CAT; EC 1.11.1.6) activity was determined on the basis of H₂O₂ decomposition at 240  nm.^38^ The reaction mixture contained 50  mM phosphate buffer (pH 7.0), 15  mM H₂O₂, and enzyme extract. The decline in absorbance at 240 nm was monitored spectrophotometrically for 1 min, and activity was calculated from the rate of H₂O₂ breakdown. All enzyme activities were expressed as units mg^–1^ protein min^–1^.

#### Statistical analysis

Statistical analyses were performed using SPSS 20.0, OriginPro 2021, and Microsoft Excel 2019. Because the experiment included three independent factors, namely melatonin, IONPs, and salinity, a three-way analysis of variance (ANOVA) was performed to evaluate the main effects of each factor and all possible two-way and three-way interactions on morphological, physiological, and biochemical parameters. This statistical approach was adopted to ensure that the complete factorial structure of the experiment was properly represented and interpreted. Before ANOVA, the assumptions of normality and homogeneity of variance were assessed using the Shapiro‒Wilk test and Levene’s test, respectively. Tukey’s honest significant difference (HSD) test was used for post hoc mean comparison at the 95% confidence level.

## Results

### Characterization of iron oxide nanoparticles

**Figure 1.**
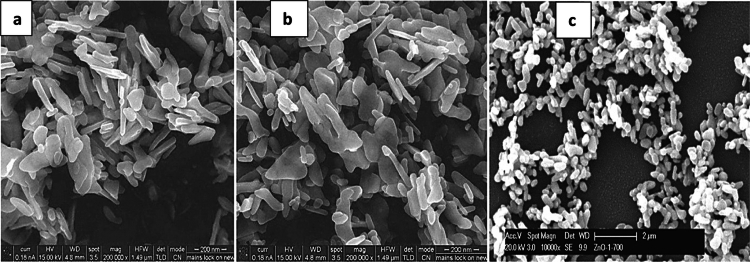
Scanning electron microscopy (SEM) images of iron oxide nanoparticles calcined at different temperatures: (a) 300 °C, (b) 500 °C, and (c) 700 °C.

**Figure 2.**
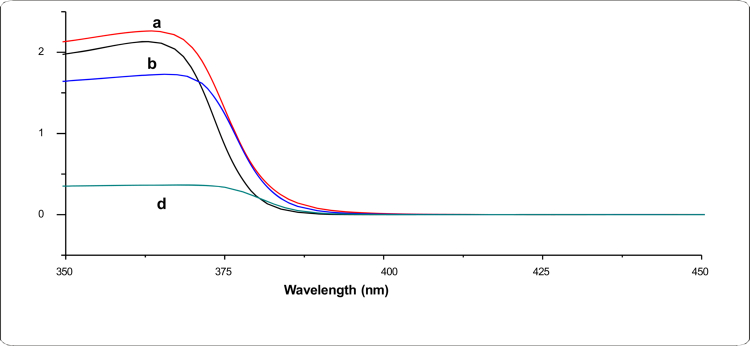
UV–Vis absorption spectra of iron oxide nanoparticles calcined at different temperatures: (a) 300 °C, (b) 500 °C, (c) 700 °C, and (d) 900 °C.

**Figure 3.**
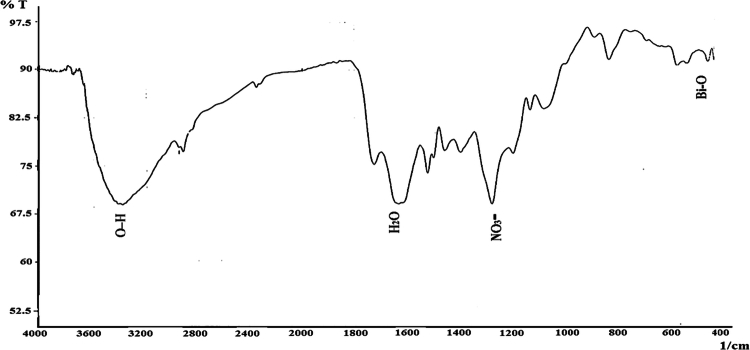
Fourier-transform infrared (FTIR) spectrum of iron oxide nanoparticles.

**Figure 4.**
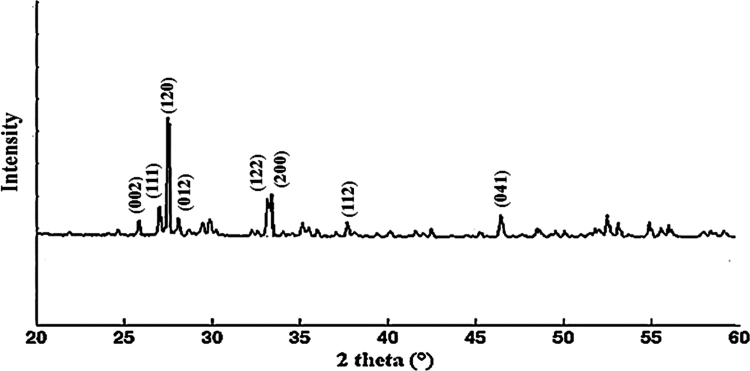
X-ray diffraction (XRD) pattern of iron oxide nanoparticles.

The synthesized iron oxide nanoparticles (IONPs) were characterized before biological application to verify their morphology, optical behavior, surface functional groups, and crystalline phase. Collectively, the characterization data confirmed successful synthesis of structurally stable iron oxide nanoparticles suitable for foliar application in peppermint. Figures 1–4 summarize the structural, spectral, and diffraction characteristics of the synthesized IONPs.

### Scanning electron microscopy (SEM)

Scanning electron microscopy showed that calcination temperature markedly affected nanoparticle morphology (Figure 1). At 300 °C, the particles appeared granular and highly aggregated, indicating incomplete crystallization and limited particle separation. At 500 °C, particle boundaries became more distinct, and aggregation decreased, suggesting improved structural organization. At 700 °C, the nanoparticles exhibited a more compact and crystalline morphology, although larger agglomerates were also visible, most likely because of sintering at the higher calcination temperature. These observations indicate that calcination temperature strongly influenced particle development, aggregation behavior, and surface texture.

### UV–vis spectroscopy

The UV‒Vis absorption spectra further supported the temperature-dependent structural changes in the synthesized nanoparticles (Figure 2). The nanoparticles calcined at 300 °C showed a broad and relatively weak absorption pattern, consistent with lower crystallinity and incomplete phase development. With increasing calcination temperature, the absorption peak became sharper and more intense, particularly at 500–700 °C, indicating improved crystallinity and enhanced electronic transitions within the iron oxide lattice. At 900 °C, a slight reduction in absorption intensity was observed, suggesting partial particle coalescence or loss of structural uniformity. Overall, intermediate calcination temperatures produced the most favorable optical characteristics.

### Fourier transform infrared (FTIR) spectroscopy

FTIR analysis confirmed the presence of characteristic functional groups associated with nanoparticle formation (Figure 3). A broad band at 3363–3414 cm^–1^ corresponded to O–H stretching vibrations, indicating surface-bound hydroxyl groups and adsorbed moisture. The band at 2330 cm^–1^ was attributed to environmental CO₂ absorption, while the peak at 1629 cm^–1^ corresponded to H₂O bending vibrations. The absorption band at 1261 cm^–1^indicated nitrate-related residues, which were likely derived from the precursor solution. Most importantly, the distinct band at 542 cm^–1^ corresponded to Fe–O vibrations, confirming the successful formation of iron oxide nanoparticles.

### X-ray diffraction (XRD) analysis of IONPs

The X-ray diffraction pattern showed sharp and well-defined peaks corresponding to the Fe₃O₄ phase (Figure 4). The characteristic reflections observed at 2θ values of approximately 30.1°, 35.6°, 43.3°, 53.8°, and 62.5° confirmed the crystalline nature of the synthesized nanoparticles. The absence of additional impurity peaks indicated good phase purity. Together, the SEM, UV–vis, FTIR, and XRD results confirmed the successful synthesis of iron oxide nanoparticles with suitable physicochemical properties for subsequent biological application.

### Effect of melatonin and IONPs on morphological and growth attributes of peppermint

The present study examined the effects of melatonin and IONPs, applied individually and in combination, on the salt-mediated stress response of peppermint. Morphological assessment showed that salinity markedly suppressed plant growth, whereas melatonin and IONPs improved plant performance under both non-saline and saline conditions. In most growth-related traits, the combined application of melatonin and IONPs produced the strongest positive response, indicating a greater protective effect than either treatment alone.

### Leaf traits

**Figure 5.**
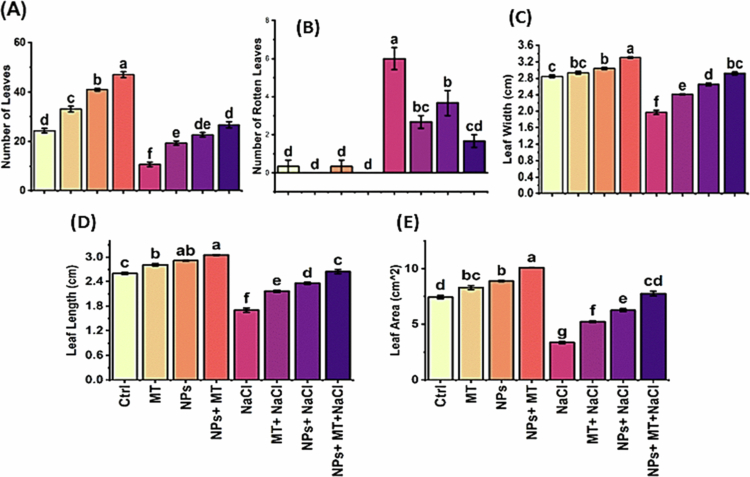
Effects of melatonin and iron oxide nanoparticles on leaf growth parameters of *Mentha piperita* under normal and saline conditions: (A) number of leaves, (B) number of rotten leaves, (C) leaf width (cm), (D) leaf length (cm), and (E) leaf area (cm²). The data are presented as mean ± SD of three biological replicates (*n* = 3). Different lowercase letters above the bars indicate significant differences among treatment means according to Tukey’s HSD test at p ≤ 0.05 following three-way ANOVA. Ctrl, control; MT, 0.1 g L^−1^ melatonin; NPs, 0.5 g L^−1^ iron oxide nanoparticles; NPs + MT, combined application of 0.5 g L^−1^ iron oxide nanoparticles and 0.1 g L^−1^ melatonin; NaCl, 100  mM NaCl; MT + NaCl, 0.1 g L^−1^ melatonin under salt stress; NPs + NaCl, 0.5 g L^−1^ iron oxide nanoparticles under salt stress; NPs + MT + NaCl, combined application of 0.5 g L^−1^ iron oxide nanoparticles and 0.1 g L^−1^ melatonin under salt stress.

Leaf-related parameters were significantly influenced by treatment (Figure 5A−E). Salinity reduced leaf production and leaf expansion while increasing leaf injury, as reflected by the higher number of rotten leaves under NaCl treatment. In contrast, melatonin and IONPs each improved leaf development, and their combined application produced the most favorable response. The number of leaves, leaf width, leaf length, and leaf area all differed significantly among treatments, with F-values of 147.35, 151.58, 183.52, and 249.42, respectively (*p* < 0.0001 in all cases). The plants receiving the combined  + melatonin treatment showed the highest leaf number (47.00 ± 2.00) and largest leaf area (10.10 ± 0.03), whereas NaCl alone resulted in the lowest leaf area (3.35 ± 0.17). Similarly, the number of rotten leaves varied significantly (F = 30.55, *p* < 0.0001), with the highest damage under NaCl alone (6.00 ± 1.00) and a marked reduction under IONPs + melatonin + NaCl (1.67 ± 0.58). These results indicate that melatonin and IONPs effectively alleviated salt-induced inhibition of leaf growth and reduced visible tissue injury.

### Shoot traits

**Figure 6.**
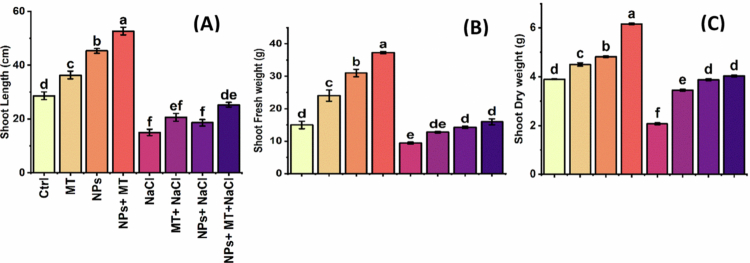
Effects of melatonin and iron oxide nanoparticles on shoot growth parameters of *Mentha piperita* under normal and saline conditions: (A) shoot length (cm), (B) shoot fresh weight (g), and (C) shoot dry weight (g). The data are presented as mean ± SD of three biological replicates (*n* = 3). Different lowercase letters above the bars indicate significant differences among treatment means according to Tukey’s HSD test at p ≤ 0.05 following three-way ANOVA. Ctrl, control; MT, 0.1 g L^−1^ melatonin; NPs, 0.5 g L^−1^ iron oxide nanoparticles; NPs + MT, combined application of 0.5 g L^−1^ iron oxide nanoparticles and 0.1 g L^−1^ melatonin; NaCl, 100  mM NaCl; MT + NaCl, 0.1 g L^−1^ melatonin under salt stress; NPs + NaCl, 0.5 g L^−1^ iron oxide nanoparticles under salt stress; NPs + MT + NaCl, combined application of 0.5 g L^−1^ iron oxide nanoparticles and 0.1 g L^−1^ melatonin under salt stress.

Shoot growth followed the same general pattern (Figure 6A–C). Salt stress caused pronounced reduction in shoot length, shoot fresh weight, and shoot dry weight, confirming the strong inhibitory effect of salinity on aerial biomass accumulation. The application of melatonin or IONPs partially counteracted this suppression, whereas the combined treatment produced the greatest recovery. Under non-saline conditions, the IONPs + melatonin treatment produced the highest shoot growth, with shoot length reaching 52.7 ± 2.5  cm, shoot fresh weight 37.3 ± 0.6 g, and shoot dry weight 6.15 ± 0.05 g. In contrast, NaCl alone reduced these values to 15.0 ± 2.0  cm, 9.4 ± 0.5 g, and 2.1 ± 0.05 g, respectively. Under saline conditions, the combined IONPs + melatonin treatment substantially improved shoot performance, with shoot length increasing to 25.3 ± 1.5  cm, shoot fresh weight to 16.6 ± 1.4 g, and shoot dry weight to 4.03 ± 0.08 g. Thus, the combined treatment was more effective than either treatment alone in sustaining shoot growth under stress.

### Root traits

**Figure 7.**
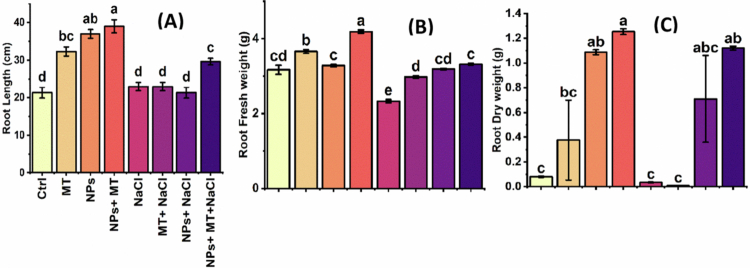
Effects of melatonin and iron oxide nanoparticles on root growth parameters of *Mentha piperita* under normal and saline conditions: (A) root length (cm), (B) root fresh weight (g), and (C) root dry weight (g). Data are presented as mean ± SD of three biological replicates (*n* = 3). Different lowercase letters above the bars indicate significant differences among treatment means according to Tukey’s HSD test at p ≤ 0.05 following three-way ANOVA. Ctrl, control; MT, 0.1 g L^−1^ melatonin; NPs, 0.5 g L^−1^ iron oxide nanoparticles; NPs + MT, combined application of 0.5 g L^−1^ iron oxide nanoparticles and 0.1 g L^−1^ melatonin; NaCl, 100  mM NaCl; MT + NaCl, 0.1 g L^−1^ melatonin under salt stress; NPs + NaCl, 0.5 g L^−1^ iron oxide nanoparticles under salt stress; NPs + MT + NaCl, combined application of 0.5 g L^−1^ iron oxide nanoparticles and 0.1 g L^−1^ melatonin under salt stress.

Root traits were also strongly influenced by salinity and exogenous treatments (Figure 7A−C). NaCl reduced root length and biomass, indicating impaired belowground growth under salt stress. Melatonin and IONPs each improved root performance relative to salt-treated plants, but their combined application resulted in the strongest enhancement. Under non-saline conditions, IONPs + melatonin produced the best root growth, with root length of 39.0 ± 3.0 cm, root fresh weight of 4.18 ± 0.05 g, and root dry weight of 1.25 ± 0.08 g. Under NaCl alone, these parameters declined markedly, with root length of 23.0 ± 1.5 cm, root fresh weight of 2.3 ± 0.2 g, and root dry weight of 0.04 ± 0.01 g. The combined IONPs + melatonin + NaCl treatment provided the strongest recovery under salinity, increasing root length to 29.7 ± 1.5 cm, root fresh weight to 3.31 ± 0.1 g, and root dry weight to 1.12 ± 0.05 g. These data indicate substantial mitigation of salt-induced root growth inhibition by the dual treatment.

### Effect of melatonin and IONPs on physiological attributes of peppermint

**Figure 8.**
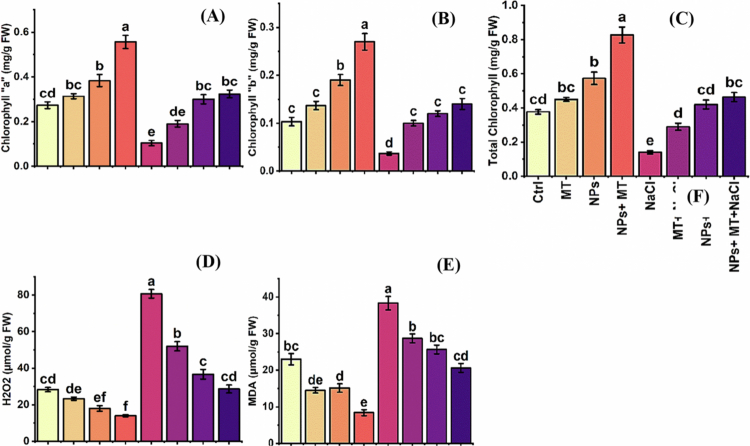
Effects of melatonin and iron oxide nanoparticles on physiological parameters of *Mentha piperita* under normal and saline conditions: (A) H₂O₂ (µmol g^−1^ FW), (B) MDA (nmol g^−1^ FW), (C) electrolyte leakage (%), (D) chlorophyll a (mg g^−1^ FW), (E) chlorophyll b (mg g^−1^ FW), and (F) total chlorophyll (mg g^−1^ FW). Data are presented as the means ± SD of three biological replicates (*n* = 3). Different lowercase letters above the bars indicate significant differences among treatment means according to Tukey’s HSD test at *p* ≤ 0.05 following three-way ANOVA. Ctrl, control; MT, 0.1 g L^−1^ melatonin; NPs, 0.5 g L^−1^ iron oxide nanoparticles; NPs + MT, combined application of 0.5 g L^−1^ iron oxide nanoparticles and 0.1 g L^−1^ melatonin; NaCl, 100  mM NaCl; MT + NaCl, 0.1 g L^−1^ melatonin under salt stress; NPs + NaCl, 0.5 g L^−1^ iron oxide nanoparticles under salt stress; NPs + MT + NaCl, combined application of 0.5 g L^−1^ iron oxide nanoparticles and 0.1 g L^−1^ melatonin under salt stress.

Physiological responses were assessed through oxidative stress markers, membrane stability, and photosynthetic pigment content (Figure 8A–F). Salinity induced severe physiological injury, as evidenced by increased hydrogen peroxide (H₂O₂), malondialdehyde (MDA), and electrolyte leakage, along with marked reductions in chlorophyll a, chlorophyll b, and total chlorophyll. In contrast, melatonin and IONPs alleviated oxidative damage and improved pigment retention, with the combined treatment having the greatest protective effect.

Under control conditions, H₂O₂, MDA, and electrolyte leakage were 28.3 ± 2.1  µmol g^–1^ FW, 23.0 ± 2.5 nmol g^−1^ FW, and 23.0 ± 1.0%, respectively. Salt stress sharply intensified oxidative damage, increasing H₂O₂ to 80.7 ± 3.8  µmol g^−1^ FW, MDA to 38.3 ± 3.1 nmol g^−1^ FW, and electrolyte leakage to 64.7 ± 4.5%. At the same time, chlorophyll content declined substantially, with total chlorophyll falling to 0.14 ± 0.02  mg g^−1^ FW under NaCl alone.

Application of melatonin or IONPs reduced oxidative stress and improved chlorophyll retention relative to salt stress alone, but the strongest protection was observed under the combined treatment. Under non-saline conditions, IONPs + melatonin reduced H₂O₂ to 14.0 ± 1.0  µmol g^–1^ FW and MDA to 8.4 ± 1.5 nmol g^–1^ FW, while increasing total chlorophyll to 0.83 ± 0.03  mg g^–1^ FW. Under saline conditions, the combined IONPs + melatonin treatment reduced H₂O₂ to 28.7 ± 2.3  µmol g^−1^ FW, MDA to 20.6 ± 2.1 nmol g^–1^ FW, and electrolyte leakage to 26.7 ± 2.0%, while restoring total chlorophyll to 0.46 ± 0.03  mg g^–1^ FW. Collectively, these results indicate that melatonin and IONPs preserved membrane integrity and photosynthetic capacity under saline conditions, with the combined treatment conferring the highest level of physiological protection.

### Effect of melatonin and IONPs on antioxidant enzyme activities

**Figure 9.**
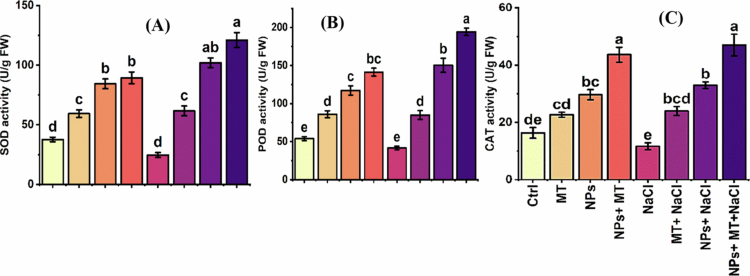
Effects of melatonin and iron oxide nanoparticles on antioxidant enzyme activities of *Mentha piperita* under normal and saline conditions: (A) superoxide dismutase (SOD) activity, (B) peroxidase (POD) activity, and (C) catalase (CAT) activity. The data are presented as mean ± SD of three biological replicates (*n* = 3). Different lowercase letters above bars indicate significant differences among treatment means according to Tukey’s HSD test at *p* ≤ 0.05 following three-way ANOVA. Ctrl, control; MT, 0.1 g L^−1^ melatonin; NPs, 0.5 g L^−1^ iron oxide nanoparticles; NPs + MT, combined application of 0.5 g L^−1^ iron oxide nanoparticles and 0.1 g L^−1^ melatonin; NaCl, 100  mM NaCl; MT + NaCl, 0.1 g L^−1^ melatonin under salt stress; NPs + NaCl, 0.5 g L^−1^ iron oxide nanoparticles under salt stress; NPs + MT + NaCl, combined application of 0.5 g L^−1^ iron oxide nanoparticles and 0.1 g L^−1^ melatonin under salt stress.

The activities of the key antioxidant enzymes superoxide dismutase (SOD), peroxidase (POD), and catalase (CAT) were significantly influenced by treatment (Figure 9). In general, melatonin and IONPs enhanced antioxidant enzyme activity, whereas NaCl alone caused an overall decline in enzymatic defense. The combined treatment produced the most pronounced stimulation of the antioxidant system.

Under control conditions, SOD, POD, and CAT activities were 37.7 ± 3.1, 54.0 ± 4.5, and 16.3 ± 3.2 U mg^–1^ protein, respectively. Salt stress reduced these values to 24.7 ± 3.6, 41.7 ± 4.2, and 11.7 ± 2.1 U mg^–1^ protein, respectively. In contrast, the exogenous application of melatonin and IONPs enhanced antioxidant enzyme activity under both normal and saline conditions. The combined IONPs + melatonin treatment produced the highest enzyme activities under non-saline conditions, with SOD, POD, and CAT reaching 89.3 ± 8.5, 141.3 ± 9.2, and 43.7 ± 4.5 U mg^–1^ protein, respectively. Under salinity, the strongest enzymatic response was again recorded for the combined treatment, where the SOD activity increased to 121.0 ± 7.3 U mg^–1^ protein, the POD to 194.3 ± 8.5 U mg^–1^ protein, and the CAT to 47.0 ± 6.1 U mg^–1^ protein. These findings indicate that the protective effects of melatonin and IONPs against salinity are closely associated with enhanced enzymatic scavenging of reactive oxygen species.

Since *M. piperita* is primarily cultivated for essential oil production, evaluation of essential oil yield, menthol content, and oil composition would further strengthen the practical relevance of this study. These parameters were not assessed in the present work and should be considered in future investigations.

## Discussion

The present study systematically evaluated the individual and synergistic effects of iron oxide nanoparticles (IONPs) and melatonin on salinity-induced stress responses in *Mentha × piperita* by integrating morphological, physiological, and biochemical analyses. The response variables assessed included vegetative growth traits, photosynthetic pigment content, oxidative stress markers, membrane injury, and the activities of the major antioxidant enzymes superoxide dismutase (SOD), peroxidase (POD), and catalase (CAT). Across these response domains, salinity imposed a clear inhibitory effect, whereas melatonin and IONPs alleviated stress injury to varying degrees. Importantly, their combined application consistently produced the strongest response, indicating that these two inputs act in a complementary, and likely synergistic, manner to improve peppermint performance under saline conditions.

Salinity is a complex abiotic stress that imposes both osmotic and ionic constraints on plant function. The initial reduction in external water potential restricts water uptake and cell expansion, whereas the subsequent accumulation of toxic ions, particularly Na⁺ and Cl⁻, disrupts ion homeostasis, membrane function, nutrient acquisition, and metabolic organization. These primary effects are commonly followed by secondary oxidative stress arising from the overproduction of reactive oxygen species in chloroplasts, mitochondria, peroxisomes, and plasma membrane-associated redox systems.^1-15^ The present results fit this established framework. Under NaCl treatment, peppermint exhibited depressed leaf development, reduced shoot and root growth, severe declines in chlorophyll a, chlorophyll b, and total chlorophyll, and marked increases in hydrogen peroxide, malondialdehyde, and electrolyte leakage. Taken together, these changes indicate coordinated deterioration of growth, photosynthetic competence, membrane stability, and redox balance under salt stress.

A central outcome of this work was the clear protective role of IONPs. Iron is a functionally indispensable micronutrient in higher plants because it is involved in chlorophyll biosynthesis, photosynthetic electron transport, mitochondrial respiration, Fe‒S cluster formation, and the catalytic activity of a wide range of oxidoreductases. Under saline conditions, Fe acquisition and translocation are often impaired owing to rhizosphere chemical changes, competitive ion effects, and generalized disruption of nutrient uptake systems. This may intensify chlorosis, impair photosynthetic metabolism, and aggravate oxidative imbalance. In this context, nanoscale iron delivery may offer physiological advantages over conventional inputs because of its high surface area, enhanced reactivity, and potentially improved interactions with plant surfaces and tissues.^7^^,^^8^^,^^27-29^ In the present study, IONP-treated plants showed better chlorophyll retention, greater biomass accumulation, and lower oxidative injury than salt-stressed untreated plants, supporting the view that iron-based nanomaterials help preserve central metabolic functions under salinity stress.

The protective effect of melatonin was likewise evident and agrees with its well-established role as a pleiotropic regulator of plant stress adaptation. Melatonin functions both as a direct antioxidant and as an upstream regulator of stress-responsive pathways, influencing redox homeostasis, osmotic adjustment, membrane protection, stomatal behavior, and phytohormonal signaling.^17-25^ In the present experiment, melatonin reduced hydrogen peroxide, malondialdehyde, and electrolyte leakage while improving chlorophyll content and growth traits under saline conditions. These results are consistent with reports in rice, wheat, tomato, and other crop species in which exogenous melatonin improved chloroplast stability, restricted lipid peroxidation, and enhanced growth under salinity.^18^^,^^21-25^ Thus, the peppermint response observed here is mechanistically congruent with a broader body of evidence showing that melatonin strengthens plant tolerance primarily through stabilization of the cellular redox status and maintenance of photosynthetic functionality.^17-25^

The most important finding of the present work, however, was the superior performance of the combined melatonin + IONP treatment. Relative to single treatments, the dual application produced the greatest improvements in leaf area, shoot and root development, chlorophyll preservation, oxidative stress reduction, and antioxidant enzyme activity, especially under NaCl stress. This pattern strongly suggests complementarity between the physiological actions of melatonin and the metabolic support provided by iron nanoparticles. Recent studies aligned with the present system support this interpretation. In peppermint, exogenous melatonin in the presence of Fe₃O₄-based nanomaterials has been reported to improve biomass-related and biochemical traits, indicating that aromatic plants can respond favorably to such combined interventions.^26^^,^^32^ Similarly, Askary et al.^16^ demonstrated beneficial effects of iron nanoparticles on *M. piperita* under salinity, while more recent work has shown that melatonin can modify biomass accumulation and biochemical metabolism in peppermint exposed to iron-based nanomaterials.^26^ The present data extend these observations by showing that the combined treatment is not only beneficial under non-stress conditions but also especially effective in counteracting salt-induced injury.^30^

From a mechanistic perspective, the dual effects of melatonin and IONPs can be interpreted at several interconnected levels. First, the improved chlorophyll retention suggested protection of chloroplast structure and function. Since chlorophyll loss under salinity is commonly associated with oxidative damage, impaired Fe-dependent biosynthesis, and degradation of pigment‒protein complexes, the relatively high chlorophyll levels recorded in treated plants likely reflect both improved Fe availability and a relatively low oxidative burden.^3-5^^,^^18^^,^^27^^,^^28^ Second, the reduction in hydrogen peroxide and malondialdehyde levels indicates more efficient control of ROS accumulation and reduced peroxidative damage to membrane lipids. Third, the lower electrolyte leakage observed in treated plants implies better maintenance of membrane selectivity and structural integrity, which is essential for sustaining ion gradients, metabolite compartmentation, and cellular viability. These biochemical effects provide a coherent explanation for the observed restoration of growth under stress.

The antioxidant enzyme data further strengthen this interpretation. SOD, POD, and CAT constitute a major enzymatic detoxification network in plant cells. SOD catalyzes the dismutation of superoxide radicals into hydrogen peroxide, while POD and CAT subsequently detoxify hydrogen peroxide through complementary reaction systems. The decline in antioxidant activity under NaCl alone in the present study reflects an impaired defensive state in severely stressed plants. In contrast, melatonin and IONPs, particularly in combination, strongly stimulated SOD, POD, and CAT activity. This enhancement implies that the treated plants had a greater capacity to intercept ROS before they could trigger extensive membrane damage and metabolic disruption. Comparable stimulation of antioxidant enzymes has been reported in many salt-stressed plant systems exposed to melatonin or nanomaterials, indicating that activation of the antioxidant machinery is a conserved component of stress mitigation.^32^ In peppermint, the magnitude of enzymatic enhancement under the combined treatment provides strong biochemical evidence that ROS detoxification is a major basis of the observed salt tolerance.

The physiological significance of improved root growth should also be emphasized. Root systems are primary sites of salt perception, ion uptake, and osmotic adjustment. Under salinity, the inhibition of root elongation and biomass accumulation reduces water acquisition, nutrient uptake, and the plant’s ability to sustain shoot metabolism.^2-5^^,^^15^ In the present study, NaCl strongly suppressed root growth, whereas melatonin and IONPs partially restored it, with the combined treatment producing the greatest recovery. This finding is important because improved root development under salinity likely enhances the overall functional capacity of the plant, not only by supporting water and mineral uptake but also by improving the plant’s ability to maintain growth despite ionic stress. Thus, the beneficial effect of the treatments should be viewed as operating at the whole-plant level rather than as a purely leaf-based antioxidant response.

Although ion transporter expression and tissue ion concentrations were not directly quantified in the present work, the results are compatible with improved ionic homeostasis in treated plants. Salinity tolerance depends not only on antioxidant capacity but also on the ability to restrict Na⁺ toxicity, preserve K⁺ retention, and maintain membrane transport processes. Melatonin has been implicated in the regulation of ion transport, vacuolar sequestration, and osmotic adjustment,^21^^,^^24^^,^^25^ whereas iron availability influences energy metabolism and membrane-associated transport competence.^7^^,^^8^^,^^28^ The combined treatment may therefore improve physiological performance through a dual effect on redox buffering and ion-management processes. This interpretation is consistent with the overall pattern of improved chlorophyll retention, lower membrane leakage, better root growth, and reduced oxidative injury observed in the treated plants.

The present results are especially relevant because peppermint is a high-value medicinal and aromatic species in which abiotic stress may affect not only biomass but also biochemical quality. From an agronomic perspective, maintaining growth, pigment stability, and oxidative balance under salinity is likely to be advantageous for preserving productivity. At the same time, the practical significance of the current study could be strengthened further by linking these physiological gains to commercial-quality traits. In particular, essential oil yield, menthol content, and oil composition were not evaluated here, although peppermint is primarily cultivated for these attributes. Recent peppermint-based studies indicate that melatonin and Fe₃O₄-related treatments can influence biomass, carbohydrate metabolism, and essential oil production.^16^^,^^26^^,^^32^ Therefore, the incorporation of essential oil extraction and GC‒MS profiling into future work would be especially valuable for determining whether the stress-mitigating effects described here translate into improvements in product quality and industrial value.

Another important aspect of the present work is the use of biogenic iron nanoparticles. Plant-mediated synthesis offers a potentially advantageous route for agricultural nanomaterials because it may reduce the chemical burden associated with conventional nanoparticle synthesis and introduce phytochemical capping agents that influence particle stability and biological interactions.^29^^,^^31^ In this sense, the use of *M. oleifera*-derived IONPs is not only methodologically relevant but also agriculturally attractive. Such materials may combine functional efficacy with better environmental compatibility, which is an important consideration in the development of nano-enabled crop management strategies for salt-affected systems.^8^^,^^31^

Despite the clear physiological and biochemical trends documented here, some limitations should be acknowledged. First, the experiment was conducted using a single concentration of melatonin and a single concentration of IONPs. Accordingly, the optimal dose range, toxicological threshold, and shape of the dose‒response relationship remain unresolved. Since nanoparticle responses are often concentration-dependent, future work should include multiple doses to determine the threshold between stimulatory and inhibitory effects.^27-29^ Second, although the antioxidant and growth responses strongly support a protective mechanism, the intracellular mode of action was not directly probed at the molecular level. Future research should therefore focus more explicitly on mechanistic resolution, including ion transport, osmolyte metabolism, membrane lipid remodeling, Fe assimilation, ROS-signaling dynamics, and stress-responsive gene expression. Transcriptomic, proteomic, and metabolomic approaches would be especially informative in defining how melatonin and IONPs interact within tissues and cells to produce the stress-mitigating phenotype. Third, since the present work was performed under greenhouse conditions, field validation will be necessary before these treatments can be recommended for broader agronomic applications.

## Conclusion

The present study demonstrates that salinity severely impairs the growth and physiological performance of *Mentha × piperita* by reducing leaf, shoot, and root development, disrupting photosynthetic pigment accumulation, increasing oxidative damage, and weakening antioxidant defense. Exogenous application of melatonin and iron oxide nanoparticles (IONPs), particularly in combination, substantially alleviated these adverse effects by enhancing chlorophyll retention, stimulating the activities of SOD, POD, and CAT, reducing hydrogen peroxide, malondialdehyde, and electrolyte leakage, and improving overall plant vigor under salt stress. The superior performance of the combined treatment indicates that melatonin and IONPs act through complementary physiological and biochemical mechanisms to strengthen stress tolerance in peppermint. These findings highlight the potential of iron-based nano-biostimulants integrated with melatonin as an effective strategy for improving plant resilience in saline environments, while also providing a strong basis for future studies on dose optimization, molecular mechanisms, essential oil-related quality traits, and field-level validation.

## Data Availability

The author confirms that all data generated or analyzed during this study are included in this published article.
